# Rapid and improved surface passivation method for Single-Molecule experiments^☆^

**DOI:** 10.1016/j.ymeth.2026.01.003

**Published:** 2026-01-07

**Authors:** Alyssa N. Gonneville, Alyssa E. Ward, Narisa Ria Naidoo, Francisco N. Barrera, Rajan Lamichhane

**Affiliations:** 1 Department of Biochemistry & Cellular and Molecular Biology, University of Tennessee, Knoxville, TN 37996, USA

**Keywords:** Single-molecule, TIRF microscopy, PEG-Silane, PEGylation, FRET, Photobleaching

## Abstract

Single-molecule fluorescence experiments are a powerful tool for studying biomolecular interactions, including protein dynamics and oligomerization, protein–protein interactions, and protein-nucleic acid interactions. Biomolecules are commonly immobilized on the microscope surface to extend the observation time. However, non-specific interactions between biomolecules and the surface present a major challenge. The first critical step in these experiments is preparing the surface using polyethylene glycol (PEG) coated slides, which facilitate biomolecule immobilization while minimizing non-specific interactions. The surface treatment typically uses PEG-SVA (Succinimidyl Valerate) coated slides, and the protocol for the treatment is lengthy and time-consuming. To overcome this issue, we have developed a process that uses PEG-Silane to improve efficiency while maintaining reproducibility. *Here*, we present a one-step, rapid PEGylation methodology that can be completed in minutes rather than hours. We demonstrate its validity and feasibility through single-molecule fluorescence resonance energy transfer (smFRET) and single-molecule photobleaching experiments across various biological samples.

## 1. Introduction

Single-molecule studies using total internal reflection fluorescence (TIRF) microscopy have become a fundamental tool for probing the assembly and dissociation of individual molecular complexes as well as for monitoring the conformational dynamics of individual molecules. An essential requirement in this approach is the efficient immobilization of individual molecules on the surface of microscope slides. A technological challenge arises due to the propensity of fluorescently labelled molecules, especially proteins, to non-specifically attach to the slide surface, producing a non-desired, bright background signal. To prevent non-specific surface interactions of proteins, meticulously cleaned quartz surfaces are usually coated with polyethylene glycol (PEG). PEG-passivated slides are routinely used in a wide variety of single-molecule fluorescence experiments, including those involving DNA, RNA, peptides, soluble proteins, and membrane proteins. The PEG-passivated surface of the microscope slides is essential, not only to prevent non-specific interactions with the slide surface, but also for immobilizing biomolecules through biotin-streptavidin interactions.

Such surface treatments typically involve multiple time-consuming reaction steps that are tedious and highly sensitive to minor variations in the protocol. *Here*, we present a straightforward single-step coating method that systematically produces high-quality PEGylated surfaces available within several minutes. The feasibility of the protocol for single-molecule fluorescence experiments has been validated through TIRF measurements of several biological samples. The results demonstrated an outstanding signal-to-noise ratio of recorded fluorescence time trajectories and negligible background, generating high-quality data.

Different approaches have been developed to coat slide/coverslip surfaces with PEG to prevent protein adsorption [1–3]. The most common surface modification strategy used in the single-molecule fluorescence experiments involves a two-step process: aminosilanization followed by PEGylation using NHS-ester chemistry [4,5]. This approach, however, is laborious and time-consuming. In this method, the quartz slide is pre-cleaned and aminosilanized, after which the primary amines are incubated for 3–4 h with a Succinimidyl Valerate (SVA)-modified methoxy polyethylene glycol (mPEG-SVA) ester. To generate a biotinylated surface for biomolecule immobilization, a small amount (2–3%) of biotin-PEG-SVA is included.

Several alternative techniques have been reported to shorten the otherwise lengthy process of cleaning and passivating microscope slides. These include treatment with 0.1% SDS to remove biomolecules and regenerate the slide chamber, as well as variations of PEG-Silane-based protocols [6–8]. While these techniques provide partial improvements, they remain relatively time-intensive and less efficient. In contrast, the new method we developed utilizes a one-minute PEGylation step (1MP). In our approach, the pre-cleaned slides are silanized with mPEG-Silane, containing a small percentage of biotin-PEG-Silane. Compared to PEG-SVA, the incubation time required for PEG-Silane is significantly reduced, enabling the preparation of large batches of slides suitable for single-molecule TIRF microscopy.

Additionally, the 1MP method ranks among the fastest available protocols for surface cleaning and regeneration compared with established PEG passivation and functionalization methods, while requiring less than 5 μM mPEG and 0.5 μM biotin-PEG (see Supplementary Table S1 for details on the cleaning protocols and PEG functionalization steps of published PEGylation procedures). For comparison, current PEGylation protocols report ~ 60 to 158 min of the preparation of new glass coverslips and slides, or up to 200 + minutes with some overnight incubations for the regeneration of prior-used slides, and use a range of 16–50 μM or higher concentrations of mPEG [5–13]. Our 1MP method offers one of the shortest cleaning times of 80 min for the preparation of new slides/coverslips with the minimum possible protocol time being 60 min. By comparison, the method published by Paul *et al.* (2021) requires a minimum of 60 min of cleaning time but suggests a range of up to 90 min for new surfaces. In addition, their protocol involves flaming the slides/coverslips, a step that must be performed with caution to avoid overheating and potential breakage. This protocol also uses PEG-SVA and, like other methods employing PEG-SVA, PEG-NHS, or PEG-SCM, requires an aminosilanization step using a mixture of 3-aminopropyltrimethoxysilane (APTES), methanol, and acetic acid. This mixture is combustible and toxic, and APTES is a potential carcinogen that requires careful handling to minimize personnel and environmental exposure [5–7,9,10,13]. An advantage of PEG-Silane-based protocols is that they eliminate the aminosilanization step altogether.

**Scheme 1.**
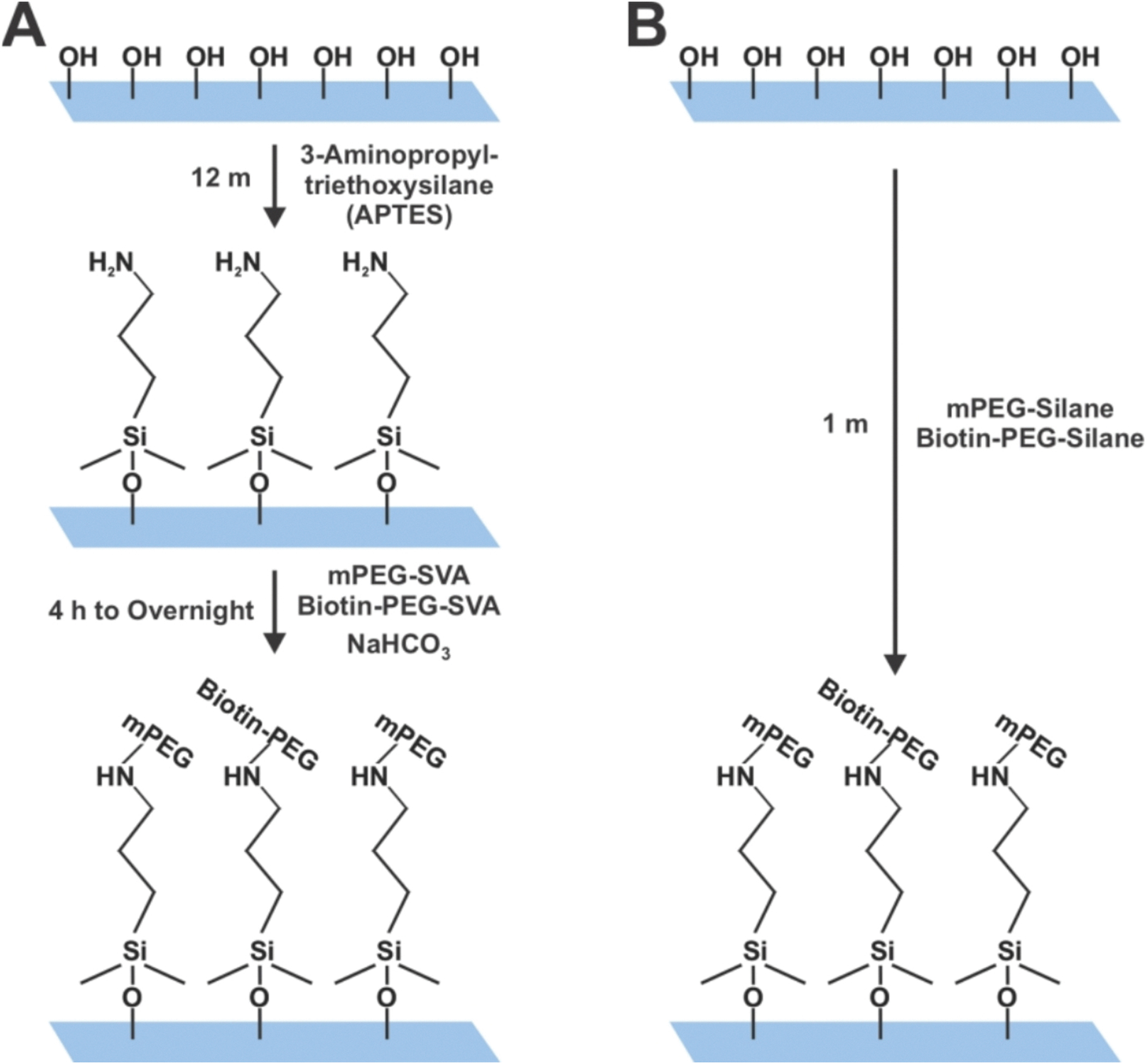
Comparison of the conventional PEGylation method and the new approach presented here. **(A)** The conventional PEGylation method is a two-step process in which the activated hydroxy groups on the pre-cleaned slides are silanized with 3-amino-propyl-trimethoxysilane (APTES), introducing primary amines. In the second step, these amines are pegylated using a PEG-SVA. The process requires 3–4 h to overnight incubation time. **(B)** The new PEGylation method consists of a single step process, where the hydroxy groups on the pre-cleaned slide are directly silanized using PEG-Silane within a minute.

In addition to the 1MP PEG-Silane method we present here, Gidi *et al.* also employ a PEG-Silane for passivation, using a PEGylation time of 15 min. In their study, the authors tested PEG-Silane incubation times ranging from 1 to 30 min and concluded that 15 min of incubation reduced non-specific binding of a small Cy3-conjugated ssDNA to their coverslips. This observation is consistent with the tendency of smaller molecules to interact non-specifically with glass surfaces that are not fully passivated by PEG. In addition, their protocol requires a custom reaction chamber for the PEGylation step that includes a desiccant, a holder, and a silicon mold defining the PEGylation area, and must be heated to 90°C. These requirements limit the number of coverslips and surface area that can be functionalized at one time. In comparison, our 1MP method can functionalize large batches of slides and coverslips (30 + pairs) as described below with minimal PEG concentrations (Supplementary Table S1). The PEG-silane method is also more cost-effective than the PEG-SVA approach, which additionally requires the purchase of APTES (Supplementary Table S1). Moreover, our 1MP method allows the slide to be regenerated up to ~ 10 times without SDS treatment, as required in some protocols. Avoiding SDS exposure reduces the risk of perturbing protein dynamics [6,7]. In summary, we present the 1MP method that reduces the time-consuming PEGylation step with the surface treatment of slides achieved in seconds (Scheme 1).

## 2. Description of methods

*Here*, we describe the protocol for a new rapid PEGylation-based slide cleaning and passivation method. We provide a detailed overview of how we prepared the slides, followed by the validation and application of our 1MP method for use in a variety of single-molecule applications.

### 2.1. Slide cleaning and passivation

Slides must be cleaned to remove any residues or debris and provide an even surface for functionalization. This process helps prevent potential artifacts that could cause background fluorescence during imaging. As published in [9,10,14], cleaning and passivation of 10–12 slides with PEG-SVA can take 14–18 h, typically 3–6 h of cleaning on the first day, followed by an overnight incubation, and an additional 1–2 h of cleaning to store slides on the following day. Outlined below is our protocol that offers both a reduced cleaning procedure and a 1-minute functionalization step. It should be noted that this protocol emphasizes the regeneration of quartz slides, as all experiments were performed on a prism TIRF system [12,14]. Quartz slides are expensive and reusing them helps reduce overall costs. Used quartz slides undergo extensive cleaning (steps 1–4) to remove tape and grease residues as well as any organic material from the slides prior to passivation. In addition, this protocol can be applied to brand-new glass or quartz slides, starting at step 5, which initiates the PEGylation functionalization process.

1. Boil pre-drilled quartz microscope slides prepared as detailed in [13] into a beaker filled with MilliQ water for 15 min. If preparing coverslips or unused surfaces for objective TIRF applications, start the cleaning procedure at Step 7.
  Notes: *Here*, we used custom 10–12 slot Teflon microscope slide/coverslip holders to secure the slides and coverslips, ensuring the surfaces remained separated for proper solvent access [13]. This boiling step is optional when using new slides but is recommended for previously used slides to help remove residual sample debris from the surface.
2. Transfer slides into a new beaker containing a 20% Contrad 70 (Decon Laboratories Inc.) solution in water and place in a water bath sonicator for 20 min.
3. After sonication, remove Contrad solution from the beaker and rinse the slides with MilliQ water three times.
4. Transfer the slides to a new beaker filled with MilliQ water and sonicate for 40 min. Then, transfer them into a glass Coplin Jar(s) for the remaining steps [5].
5. Place the Coplin Jar(s) into a water bath sonicator and add 1 M potassium hydroxide (KOH) (Sigma-Aldrich, ACS reagent pellets) to the container and sonicate for 20 min.
  Note for Objective TIRF: Place an equal number of coverslips into the Teflon holder, and soak in 1 M KOH overnight to functionalize the surfaces for the PEGylation step. Extensive KOH etching overnight significantly enhances the surface quality [9]. However, it should be noted that this could potentially introduce scratches to the glass surface. After overnight incubation in 1 M KOH, rinse the coverslips 3 times with MilliQ water, then proceed to step 9.
  Notes: This solution aids in the removal of organic material, etches the surface of the slide, and leaves it bound with hydroxyl groups (silanol) to promote subsequent functionalization [4,9,15]. KOH is classified as an irritant and corrosive material and should be handled with caution to avoid skin contact. It should be disposed of according to institutional hazardous waste procedures.
6. Rinse the slides with MilliQ water three times, then refill the Coplin Jar(s) with MilliQ water and place them back into the water bath sonicator for 40 min.
7. Next, the slides are etched in a piranha solution, consisting of 75% H_2_SO_4_ (ACS grade, Fisher Chemical) and 25% H_2_O_2_ (35 wt%, Fisher Scientific), for 20 min. Remove the Coplin Jar(s) from the water bath sonicator, decant the water, and then slowly add the piranha solution. Ensure the Coplin Jar(s) are on a level surface and can be covered after the solution is added to prevent the spreading of the solution vapor.
  Notes: This step facilitates the removal of resistant organic materials and metal contaminants [9]. This process also helps to generate a clean, highly hydroxylated surface for silanization. Caution should be taken when handling piranha solution, as the mixture can generate acute heat and vapor that are skin and lung irritants. Dispose of the piranha solution and the first water rinse of the container according to the proper hazardous waste protocol.
8. Rinse the slides with MilliQ water three times, followed by a single methanol rinse. Transfer slides back into the Teflon holders.
  Note for Prism TIRF preparation: If using new coverslips, place an equal number of coverslips in a Teflon coverslip holder and rinse once with methanol before proceeding.
9. Prepare the PEG-Silane solution by weighing out 1–2 mg of Biotin-PEG-Silane (Laysan Bio, Inc., MW 5,000) and 20–25 mg of mPEG-Silane (Laysan Bio, Inc., MW 5,000). Dissolve powders in 10 mL of acetone (Honeywell, ACS/HPLC grade, dry acetone is preferred) and 10 mL of methanol in an amber vial. *Vortex* until dissolved.
  Note: This stock solution can be stored at −20°C, sealed with parafilm, for up to one week and can be used to PEGylate up to five batches of 10–12 slide-coverslip pairs. Store the solution protected from light. If the solution becomes cloudy upon reuse, indicating degradation, prepare a fresh solution.
10. Add 4 mL of the stock PEG-Silane solution to 200 mL of acetone in a tall glass beaker.
11. Prepare a separate beaker with 200 mL of acetone (dry acetone is preferred) alone for the rinse step.
12. Dip the Teflon holder with the cleaned slides and coverslips into the beaker containing the PEG-Silane solution in acetone for 1 min, then transfer it to the rinse beaker (containing only acetone) for 30 s. Ensure the slides are fully submerged in the solution or that the solution covers the entire surface between the pre-drilled areas.
  Note: If separately cleaning coverslips, this step can be repeated using the same solutions once. If preparing multiple sets of slides/coverslips, replace the PEG/acetone solution and rinse the beaker after each slide/coverslip pair is run through the PEGylation and rinse steps.
  Note: This step differs from the commonly used functionalization method described in references [5–7,9,13], in which PEG-SVA in sodium bicarbonate buffer is applied onto an aminosilanized slide and covered with a paired coverslip, followed by incubation for a minimum of 4 h to 16 h in a light-protected humidity chamber. In contrast, our method eliminates the need to apply the solution between a slide and a coverslip, a process that can introduce air microbubbles. Additionally, the humidity can vary across chambers depending on their size, water content, and the number of slides/coverslips functionalized per container. Instead, our approach enables simultaneous functionalization of the entire batch of slides under identical conditions, thereby improving coating consistency with up to the preparation of 5 slide/coverslip batches per the PEG solution described above.
13. Remove the slides from the rinse beaker and dry them with an inert gas (e.g., Argon or Nitrogen).
14. Use immediately or put a slide/coverslip pair into a 50 mL Falcon tube, flush the container with inert gas, cap, and seal with parafilm. Store tubes at −20°C in containers with desiccant for up to one to three months [6,9].

### 2.2. Sample immobilization for Single-Molecule fluorescence experiments

**Fig. 1.**
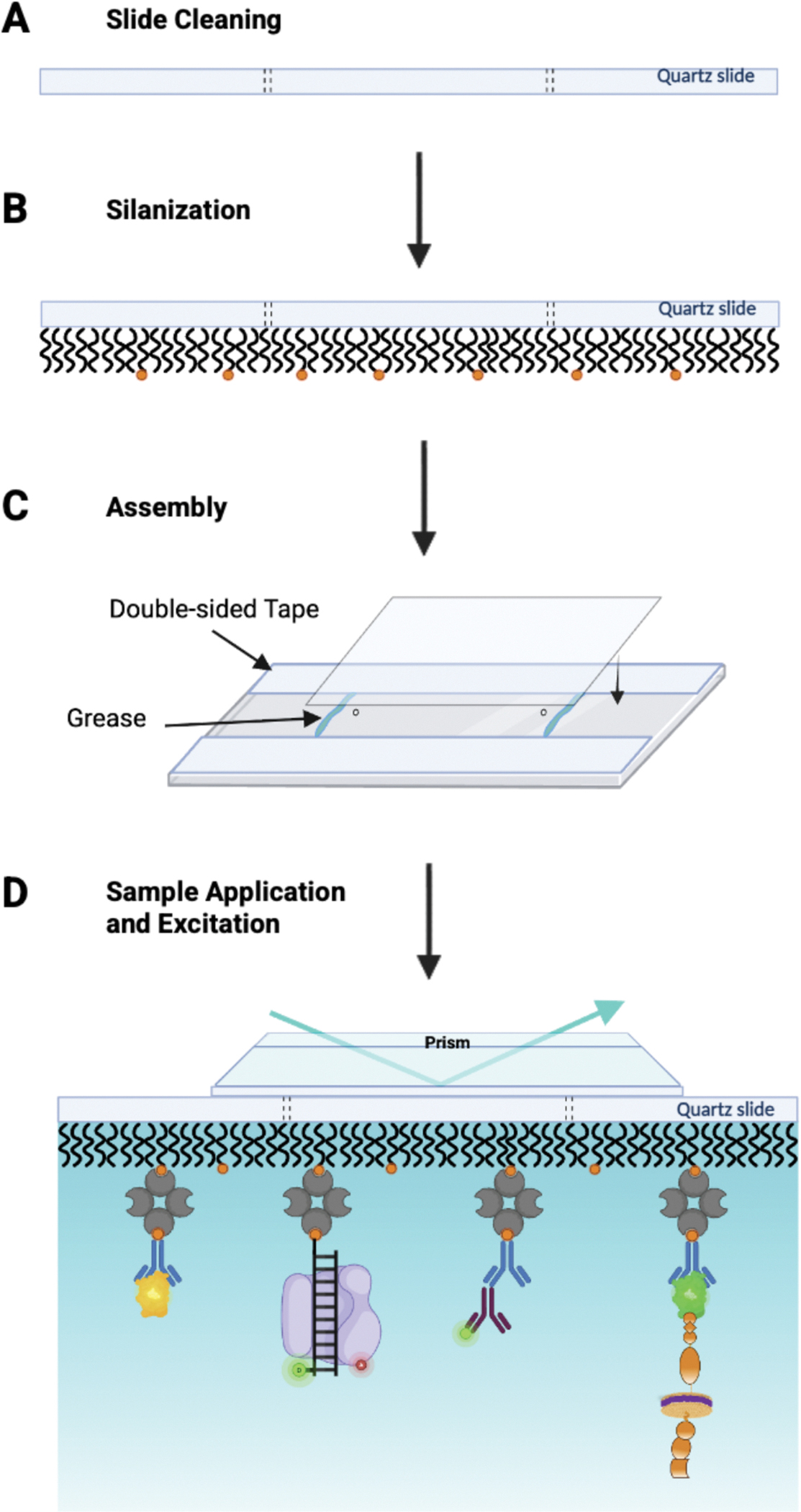
Stepwise schematic representation of slide passivation and sample application for single-molecule TIRF experiments. **(A)** The slide surface is cleaned. **(B)** PEG-Silane and biotin-PEG-Silane are conjugated to the slide surface. **(C)** Double-sided tape, grease, and a coverslip are used to create a sealed flow chamber. **(D)** The sample can be tethered using biotin-streptavidin interactions with biotinylated DNA, or biotin-conjugated antibodies, specific to the sample, such as an GFP-tagged RTK in a DIBMALP (on far right).

In this study, we performed a side-by-side comparison of PEG-SVA and PEG-Silane PEGylated quartz slides. The SVA-PEGylated slides were generated following a previously described protocol, which employed m-PEG-SVA and 3% biotin-PEG-SVA (Supplementary Table S1) [5]. Alternatively, slides were prepared using our new cleaning and passivation protocol, which utilizes mPEG-Silane and biotin-PEG-Silane (Fig. 1A & B). First, a sample chamber was prepared on the PEGylated quartz microscope slide using double-sided tape (3 M Scotch), vacuum grease (Dow Corning), and a coverslip (Corning, Rectangular #1–1/2 Cover Glass or Fisherbrand Microscope Cover Glass 24×25 mm – No. 1) (Fig. 1C) [3,13]. After forming the chamber, streptavidin (0.2 mg/mL in 10 mM Tris-HCl, pH 8.0, 50 mM NaCl buffer) was injected into the chamber, incubated for 5–10 min, and then the chamber was washed using imaging buffer (25 mM HEPES, pH 7.5, 75 mM NaCl, and 2 mM Trolox or 10 mM Tris-HCl, pH 8.0, 50 mM NaCl). The imaging buffer may vary; however, we recommend using the same buffer as in sample preparation, supplemented with 2 mM Trolox. After washing, biomolecules were immobilized via interactions with their respective biotin-conjugated antibodies, or biotinylated Ni-NTA resin, or biotinylated DNA handles (Fig. 1D) [16–20]. Once the sample was immobilized, the chamber was washed 2–3 times with imaging buffer, and an oxygen scavenging system (OSS) prepared in the same imaging buffer was added to enhance the dye stability. The OSS can vary and may consist of a simple propyl gallate solution, a glucose oxidase-catalase system (glucose, glucose oxidase, and catalase), or a PCD-PCA system (protocatechuic acid protocatechuate-3,4-dioxygenase; Sigma Aldrich/Oriental Yeast Co., respectively) [4,21–24]. All single-molecule fluorescence data were acquired using a custom-built prism-based total internal reflection fluorescence imaging system based on an inverted IX73 microscope (Olympus) equipped with a 60x water-immersion objective (NA 1.2 Olympus), as described previously [16,18,25], unless otherwise noted. A 465 nm laser (TIRF Labs) was used to excite GFP and YFP samples, and a 532 nm laser (CrystaLaser) was used to excite Alexa Fluor 555. DNA polymerase and the antibody photobleaching experiments were performed on a custom-built prism-based TIRF microscope based on an inverted Axiovert 200 (Zeiss), as described previously [25–27]. A 488 nm laser beam (Coherent) was used to excite Alexa Fluor 488, and the fluorescence emission was collected using a C-Apochromat 63x water-immersion objective (NA 1.2, Zeiss). Fluorescence emission was separated from scattered excitation light using a dichroic mirror (z488rdc, Chroma) and subsequently split into donor and acceptor detection channels, as described previously [25]. Channel separation was achieved using a Dual-View Imager (QImaging/Photometrics) equipped with a 545dcxr dichroic mirror, together with a 525/50 band-pass filter (FF02–525/50, Semrock) for the donor channel and a 620/60 band-pass filter (HQ620/60, Chroma) for the acceptor channel. Fluorescence movies were recorded using an EMCCD camera (iXon+, Andor Technology). Collected fluorescent data were processed using custom IDL software (https://github.com/Ha-SingleMoleculeLab under the Raw-Data-Analysis repository). The fluorescence intensities chosen by the IDL script are based on a previously optimized threshold. The threshold we used in the IDL script is based on the median pixel intensity (a.u.) of a 5 × 5-pixel frame, with a Standard Deviation of 25 a.u. For the FRET analysis, donor and acceptor intensity traces were background-corrected, and the acceptor signal was further corrected for donor leakage as previously described. Corrected trajectories were then used to calculate apparent FRET efficiencies using *E* = *I*_*A*_*/*_(_*I*_*D*_ + *I*_*A*_), where *I*_*D*_ and *I*_*A*_ are donor and acceptor intensities, respectively [26]. All trajectories were analyzed using custom MATLAB scripts. The photobleaching data was analyzed with a custom Python script (https://github.com/justmwest/single-molecule-photobleaching/tree/master/count_photobleaching_steps) run in the Anaconda Navigator Spyder software to identify photobleaching steps. In brief, photobleaching traces were plotted using the Spyder software script and binned into 1-step, 2-step, or ≥ 3-step categories. The step percent of each step population was calculated and graphed to represent the % step distribution of the sample based on the ratio of the accepted binned traces.

## 3. Sample preparation for Testing 1MP slides

### 3.1. A_2A_AR-Cterm peptide preparation and Purification

The human A_2A_AR C-terminus (residues 293–412) region with a site-specific cysteine (C394) for fluorophore labeling was cloned into a pET24a vector using the NdeI and XhoI cloning sites with a 6x-His tag at the C-terminus [28]. The plasmid was verified by sequencing and transformed into BL21 (DE3) cells for protein expression, as shown in the supplemental document. The cells were grown at 37°C at 225 rpm until they reached an optical density (OD_600_) between 0.6 and 0.8 in Luria-Bertani (LB) medium containing 50 μg/mL kanamycin. Protein expression was induced with 1 mM IPTG for 3–4 h at 37°C.

Cells were harvested by centrifugation at 3260 × g for 20 min at 4°C, then subsequently lysed for 1 h in a buffer containing 50 mM HEPES (pH 7.5), 500 mM NaCl, 10% glycerol (vol/vol), 1 mM TCEP, 0.1 mM PMSF, 1 μg/mL DNase I, 50 μg/mL lysozyme, and protease inhibitor (Pierce Protease Inhibitor Tablets – EDTA Free Fisher Scientific Cat #A32955). Following lysozyme treatment, the lysate was sonicated for 30 s with a 1-minute interval on ice, repeated six times to ensure total lysis. The lysate was then incubated with Ni-NTA resin overnight (16–20 h) at 4°C. The resin was washed twice with 10 column volumes (10 mL of buffer per 1 mL of resin) of wash buffer containing 50 mM HEPES (pH 7.5), 500 mM NaCl, 10% glycerol (vol/vol), 15 mM imidazole, 1 mM TCEP, and 0.1 mM PMSF. Bound protein was eluted using a gravity flow column with elution buffer consisting of 50 mM HEPES (pH 8.8), 500 mM NaCl, 10% glycerol (vol/vol), and 500 mM imidazole. The expression and purity of the peptide were monitored by SDS-PAGE (Fig. S1).

To label the A_2A_AR-Cterm peptide, an Alexa Fluor 555 maleimide dye was conjugated via thiol-reactive chemistry to a single cysteine residue in the A_2A_AR C-terminus sequence (C394). Labeling was performed at a 5:1 M ratio of dye to peptide and incubated for 2 h at 4°C. Excess free dye was removed using a desalting column. Upon labeling, we performed single-molecule fluorescence experiments by immobilizing the peptide on the microscope slide using streptavidin and a biotin-conjugated anti-histidine antibody interaction [29].

### 3.2. Cellular EphA2-GFP Extraction and DIBMALP preparation

The EphA2-GFP data were collected using the SiMPull-POP TIRF photobleaching assay, as detailed in [30] with a C-terminal GFP fused to full-length EphA2. The cell culture, treatment, and solubilization with the copolymer, diisobutylene maleic acid, to form lipid particles (DIBMALPs) are described as follows. Modified DU145 (ATCC^®^ HTB-81) mammalian cells stably expressing EphA2-GFP were maintained at 5% CO_2_ and 37°C in Dulbecco’s Modified Eagle’s Medium (DMEM) Supplemented with glucose, 10% fetal bovine serum (FBS), 100 U/mL penicillin–streptomycin, and 1 μg/mL puromycin. Cells were passaged when they reached 80% confluency. DU145 cells were lysed and harvested according to the DIBMALP preparation protocol in [20,30]. In brief, cells were lysed using a series of syringe passages, followed by centrifugation to collect the cell lysate. Lysates were then ultracentrifuged to harvest the cell membrane fraction. Membrane fractions were treated with Ephrin A1-Fc, then incubated overnight with the copolymer diisobutylene maleic acid to form DIBMALPs. Samples were ultracentrifuged to separate the solubilized DIBMALPs from unsolubilized material. The GFP fluorescence of the DIBMALP samples was quantified by a calibration curve based on known concentrations of purified GFP (Thermo Fisher Scientific). DIBMALPs containing EphA2-GFP (≤5 nM) were immobilized by a biotinylated EphA2 antibody (Cell Signaling) and illuminated by a 465 nm laser and imaged by TIRF microscopy as described above and in [17]. Data were processed and analyzed as described in section 2.4.

### 3.3. GFP and YFP sample preparation

Purified GFP and YFP proteins (generous gift from the Millar Lab at The Scripps Research Institute) (15–20 nM) were immobilized on prepared PEGylated slides by a biotinylated GFP antibody (20 nM) (Rockland Immunochemical Inc.). Samples were illuminated by a 465 nm laser and imaged by the TIRF microscope detailed above. The collected fluorescent data were processed and analyzed as described in section 2.4.

## 4. Validation of 1 MP PEGylation procedure

Both slide passivation methods, PEG-SVA and PEG-Silane, have been widely used to minimize non-specific interactions in single-molecule studies. These methods have proven effective across multiple classes of biological systems, ranging from nucleic acids and small peptides to full-length proteins [16–18,31–34]. *Here*, we review these experimental applications and provide evidence that the PEG-Silane method, in particular, offers a versatile and reliable approach to prevent non-specific adsorption of the biomolecules to the surface across a wide spectrum of biomolecular targets [9].

### 4.1. Comparison of PEG-SVA and PEG-Silane slide surfaces

**Fig. 2.**
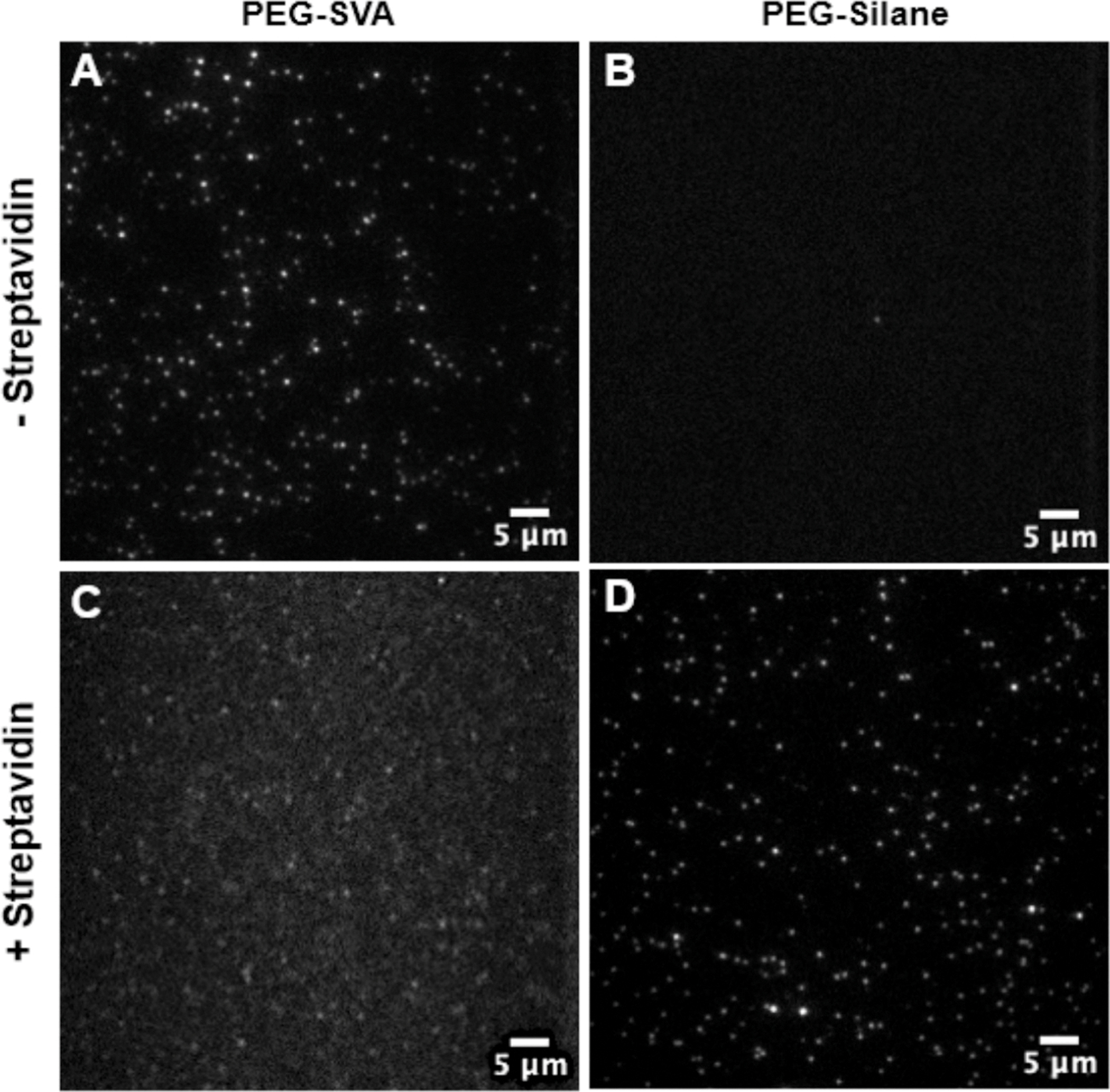
Comparison between the PEG-SVA and PEG-Silane (1MP) passivation methods using the small A_2A_AR-Cterm peptide (125 nM) labeled with the Alexa Fluor 555. **(A)** A_2A_AR-Cterm was added to the slide without streptavidin for immobilization and washed on the PEG-SVA slide (240.0 ± 100.7 s.d.). **(B)** A_2A_AR-Cterm added to 1MP slide and washed without streptavidin for immobilization (4.3 ± 2.9 s.d.). **(C)** A_2A_AR-Cterm immobilized on PEG-SVA slide surface with streptavidin (209.3 ± 84.5 s.d.). **(D)** A_2A_AR-Cterm immobilized on 1MP prepared slide with streptavidin (218.2 ± 82.3 s.d.).

To test the surface-passivation of the newly proposed 1MP silane slides, we conducted control experiments using a peptide portion of the human A_2A_ adenosine receptor’s helix 8 and C-terminal tail (A_2A_AR-Cterm), that is mainly disordered and carries a positive charge. In the experiments, we tested the labeled peptide on both the PEG-SVA and PEG-Silane slides in the presence and absence of streptavidin. A_2A_AR-Cterm was incubated with a biotin-conjugated anti-histidine antibody on the slide for 15 min, with and without streptavidin, followed by washing with imaging buffer. In the absence of streptavidin on PEG-SVA slides, the average molecule count was 240.0 ± 100.7 s.d. per field-of-view of approximately 9332 μm^2^ (Fig. S4, Supplementary Table S3). By comparison, PEG-Silane slides exhibited only 4.3 ± 2.9 s.d. molecules per field-of-view (Fig. 2A & B, Supplementary Table S3). This suggests that PEG-Silane surface passivation produces a more uniform coating, which reduces the surface available for proteins to interact non-specifically. In contrast, PEG-SVA-coated slides may allow small peptides to bind non-specifically to the quartz slide. As PEG-SVA protocols require placing the coverslip over the PEG-SVA solution on the slide and incubation, this could potentially lead to the formation of air microbubbles, resulting in areas on the slide surface that are not uniformly passivated. In the presence of streptavidin for specific immobilization of the peptide, A_2A_AR-Cterm appeared to adsorb to the PEG-SVA slide, leading to aggregation and excess background fluorescence (Fig. 2C, S2, Supplementary Table S3). However, when the process was repeated on the PEG-Silane slide, A_2A_AR-Cterm was pulled down and immobilized without excess background fluorescence (Fig. 2D, S3, Supplementary Table S3). Given that the 1MP protocol calls for slow, careful dipping of the slides into the PEG-Silane and acetone solution, it prevents the formation of air microbubbles that can occur when a coverslip is placed on the slide.

*Here*, the 1MP PEG-Silane prepared slides exhibited a reduced fluorescence background and minimized non-specific interactions with the microscope slide, providing a more uniform PEGylated coating compared to the PEG-SVA coated slides. Moreover, the 1MP protocol facilitated the immobilization of proteins that have a higher propensity to aggregate and adhere to the slide surface.

### 4.2. Immobilization and detection of labeled antibodies on 1MP surface

**Fig. 3.**
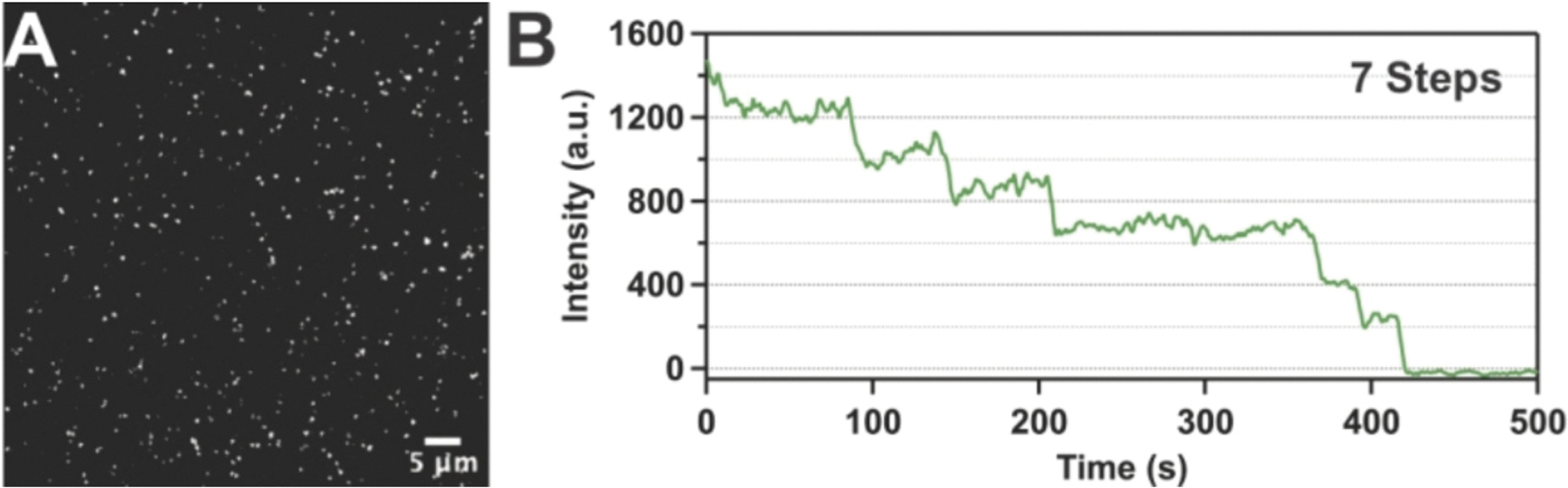
Detection of fluorescent photobleaching on 1MP slides with labeled antibodies. **(A)** TIRF image showing fluorescent spots produced by Alexa Fluor 555 labeled secondary antibodies (250 pM) bound to immobilized primary mouse anti-His antibodies. **(B)** An individual time trace shows seven discrete bleaching steps, corresponding to number of Alexa Fluor 555 dyes conjugated to a single antibody.

In the following test, we verified the quality of 1MP slides for a single-molecule pull-down (SiMPull) assay. The growing trend of this single-molecule technique is important for characterizing biomolecular complexes and interactions directly in their near-native environment [30,35–38]. In this approach, specific antibodies (biotin-conjugated) attached to the surface, via biotin-streptavidin interactions, are used to capture target proteins of interest, either purified and labeled or directly extracted from cell lysates. The treated slide surface must display robust repulsion of the many other non-specific proteins present in the sample. In this test experiment, we evaluated the Alexa Fluor 555-labeled antibody by counting photobleaching events. First, we immobilized a biotin-conjugated mouse anti-His primary antibody on the slide surface and washed with imaging buffer. Next, we incubated an Alexa Fluor 555-labeled secondary anti-mouse antibody (Thermo Fisher Scientific) for 10 min, followed by thorough rinsing. Alexa Fluor 555-conjugated antibody (250 pM) incubated on the slide in the absence of the primary antibody showed a low level of background binding (19.2 ± 8.2 mean ± s.d.) molecules per field-of-view (Fig. S5, Supplementary Table S3) compared to immobilized biotinylated primary antibody (544.8 ± 37.5) (Fig. 3A, Supplementary Table S3). According to the manufacturer’s specifications, each secondary antibody is typically labeled with 2–8 Alexa Fluor 555 dye molecules. The number of fluorescent dyes on each antibody can be determined in single-molecule imaging experiments via counting bleaching kinetics of individual dyes (each dye molecule bleaches in a single step). The data obtained demonstrate that well-fluorescent spots were detected only in the donor channel, corresponding to the binding of Alexa Fluor 555-labeled secondary antibody and producing fluorescence (Fig. 3A). The intensity jumps for individual Alexa Fluor 555 dyes were approximately 200 fluorescence arbitrary units (a.u.). The quality of the fluorescent time traces enabled us to resolve up to 7 clearly separated bleaching steps (Fig. 3B). Therefore, the data from this experiment indicate that the proposed rapid protocol provides excellent surface treatment quality for SiMPull applications.

### 4.3. Measuring the photobleaching behavior of fluorescent proteins

**Fig. 4.**
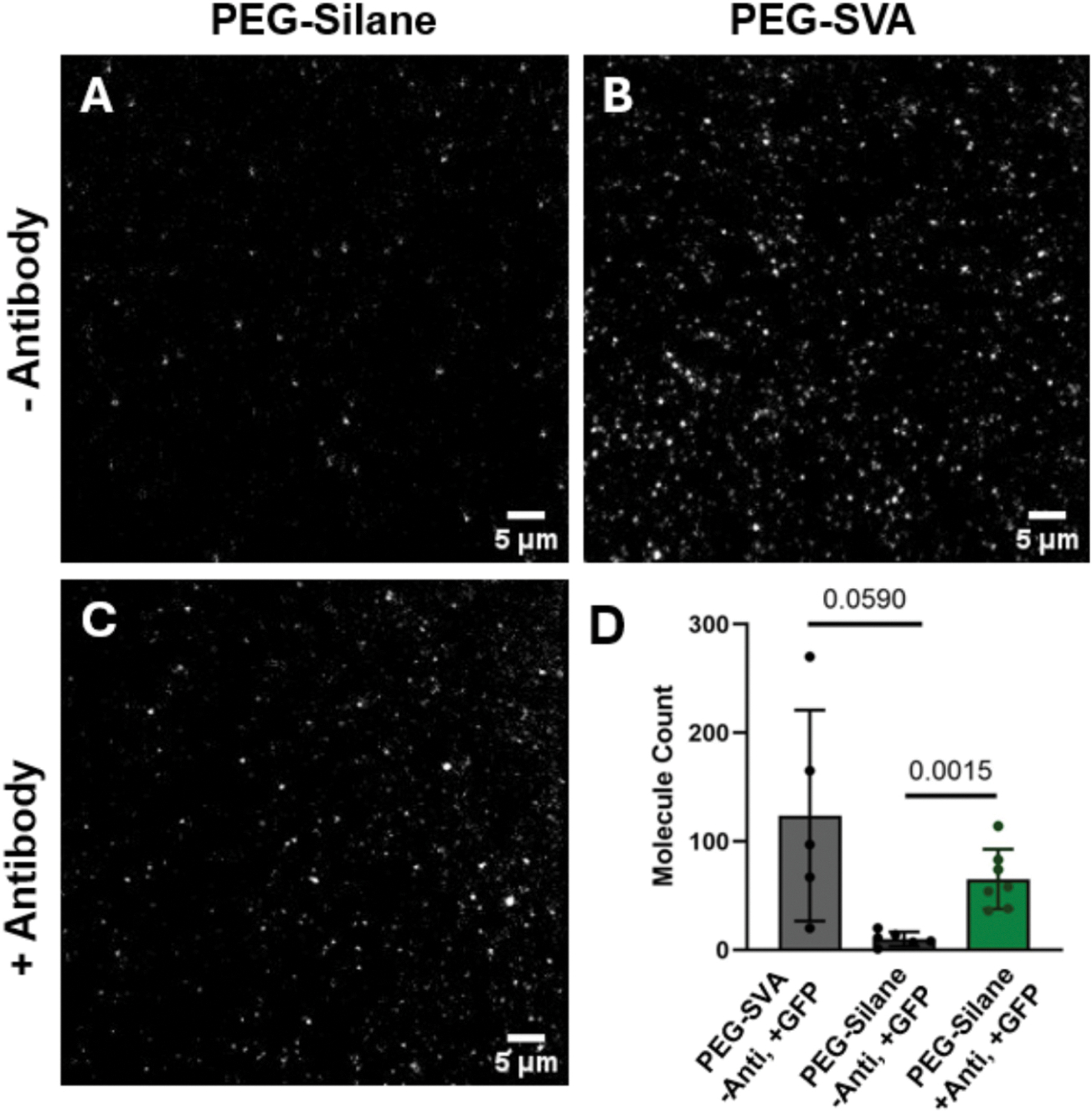
Comparison of 1MP and PEG-SVA passivated surfaces for single-molecule pull-down of a GFP. Quartz slides passivated using the 1MP method **(A)** or with PEG-SVA **(B)** incubated with NeutrAvidin and 15 nM free GFP in the absence of antibody after washing **(C)**. Antibody-immobilized GFP on a 1MP prepared slide incubated with NeutrAvidin and a biotinylated GFP antibody. **(D)** Molecule counts were obtained from 5 to 7 videos recorded from different field-of-view of the slide for each condition. Bars represent the mean ± s.d. that was calculated from the number of molecules reported per field of view for each condition (shown as individual dots). An unpaired *t*-test was run for statistical analysis to compare each data set shown.

To further assess the use of the 1MP protocol for single molecule studies, we immobilized commonly used fluorescent proteins using a biotinylated antibody and quantified their photobleaching behaviors. We first conducted control experiments to verify the potential background and non-specific binding of free GFP on slides passivated with PEG-SVA (as prepared in [9,13]) or PEG-Silane using our 1MP method. To test this, slides were prepared in the absence of the GFP antibody. They were first incubated with NeutrAvidin for 10 min, followed by a 30-minute incubation with 15 nM GFP. Five videos (representing five separate fields-of-view) were recorded per slide, and the total number of molecules per field-of-view were quantified using the custom IDL and MATLAB scripts (Fig. S6), then averaged. We observed a higher number of molecules on the slide passivated with PEG-SVA (123.8 ± 97.2 s.d., Fig. 4B, Supplementary Table S3) compared to the PEG-Silane (10.2 ± 6.5 s.d., Fig. 4A, Supplementary Table S3), even when the slide was incubated with the biotinylated GFP antibody to immobilize the GFP sample (65.3 ± 27.5 s.d., Fig. 4C & D, Supplementary Table S3), indicating potential non-specific interactions of the protein with the slide. The number of molecules quantified from the PEG-SVA slide also exhibited a larger variance, which may be due to uneven coating of the slide (Fig. 4D). This highlights the potential unequal distribution of the mPEG on the slide that acts as a brush guard against non-specific interactions and may be leaving areas for the sample to adsorb onto the slide surface. Furthermore, the uneven distribution of biotin-PEG on the passivation surface could also result in areas of low and high concentrations of the immobilized sample. This may compromise the quality of single-molecule data in highly concentrated areas, as fluorescent intensities may overlap and bias the photobleaching behavior. These results further support that our 1MP method limits non-specific interactions with the slide by providing a homogenous passivation surface that is capable of immobilizing full-length fluorescent proteins for use in single-molecule studies such as the pull-down protocol.

**Fig. 5.**
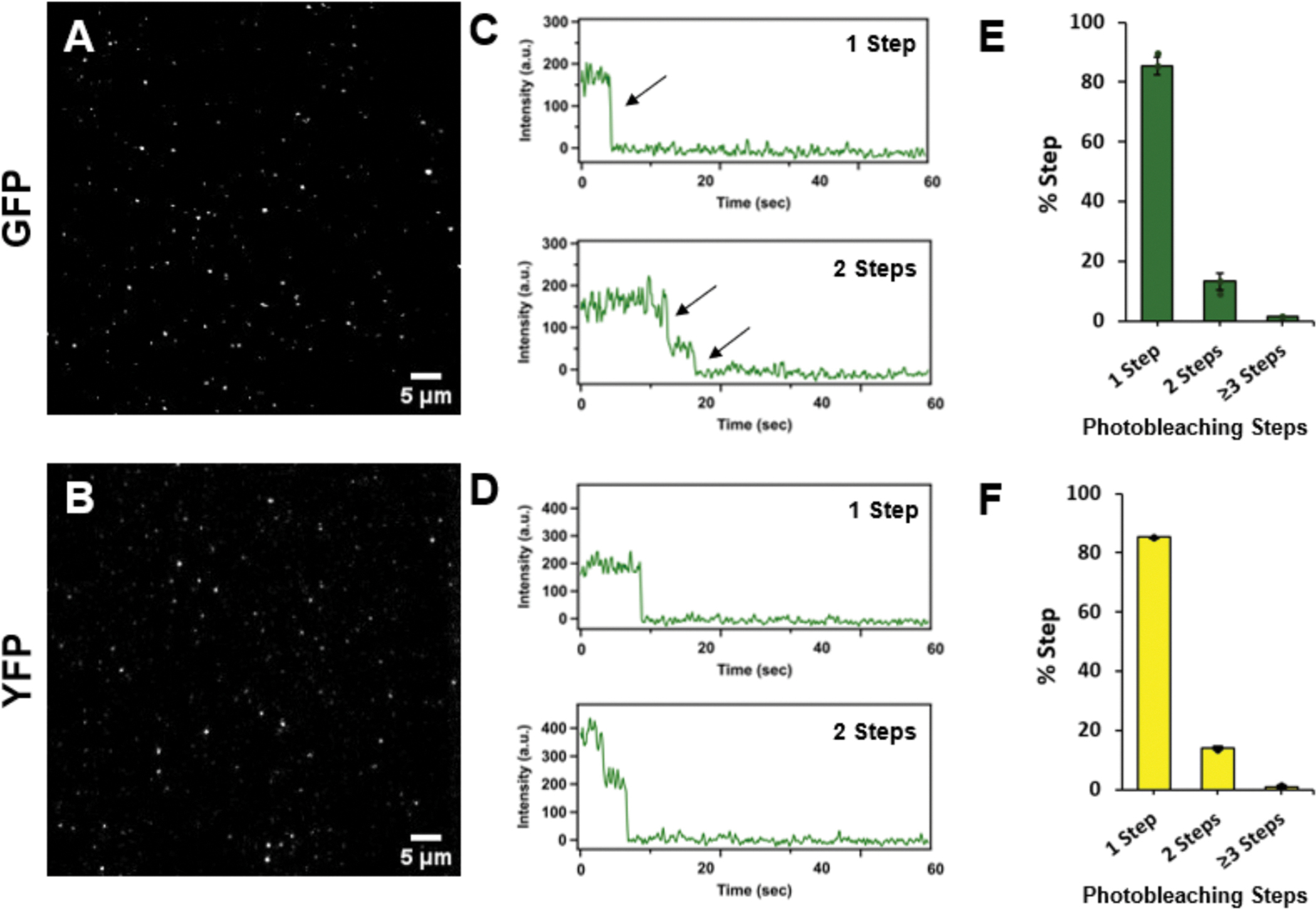
Photobleaching behavior of GFP and YFP. The fluorescent proteins (15–20 nM) were immobilized by a biotinylated GFP antibody (20 nM) on 1MP passivated slides. Representative TIRF images and photobleaching traces of immobilized GFP **(A and C)** and YFP **(B and D)**. Quantification of the percentage of 1-, 2-, and ≥ 3 photobleaching steps exhibited from immobilized GFP **(E)** and YFP **(F)**. A total of 219 molecules were analyzed for the GFP sample and 291 for the YFP sample. Bars represent mean ± s.d. Dots represent experiment replicates (N = 2).

After establishing that our 1MP method can specifically immobilize fluorescent proteins, we sought to quantify their photobleaching behavior. We recorded the fluorescent intensity (FI) of antibody-immobilized GFP (Fig. 5A) and YFP (Fig. 5B) samples over time. The number of photobleaching steps for each sample were quantified. Each FI vs time trace was individually binned as 1-, 2-, or ≥ 3 photobleaching steps for both samples (example traces are shown in Fig. 5C & D). As a control to assess non-specific binding, incubation of YFP in the absence of the biotinylated antibody used for protein immobilization (Fig. S7) resulted in significantly fewer molecules than in its presence. It is noted that the immobilization efficiency of the YFP and GFP proteins was relatively low, which may be due to antibody specificity and its ability to effectively recognize and bind these samples. Both GFP and YFP exhibited a high degree of 1-step photobleaching (85.5 ± 2.9% and 85.3 ± 0.1%, respectively) with lower amounts of 2-step traces (13.2 ± 2.9% and 14.0 ± 0.4%, respectively) and minimal to no ≥ 3 steps traces (1.4 ± 0.0% and 0.7 ± 0.5% respectively) (Fig. 5E & F). These results agree with the expected outcome that wild-type fluorescent proteins are typically monomeric but exhibit minor populations of oligomers [39–44]. This demonstrates that our 1MP method is a reliable and low-background approach for single-molecule fluorescent studies.

## 5. Applications of 1MP surface passivation

### 5.1. FRET analysis of DNA–Protein interactions and DNA polymerase I Activity

**Fig. 6.**
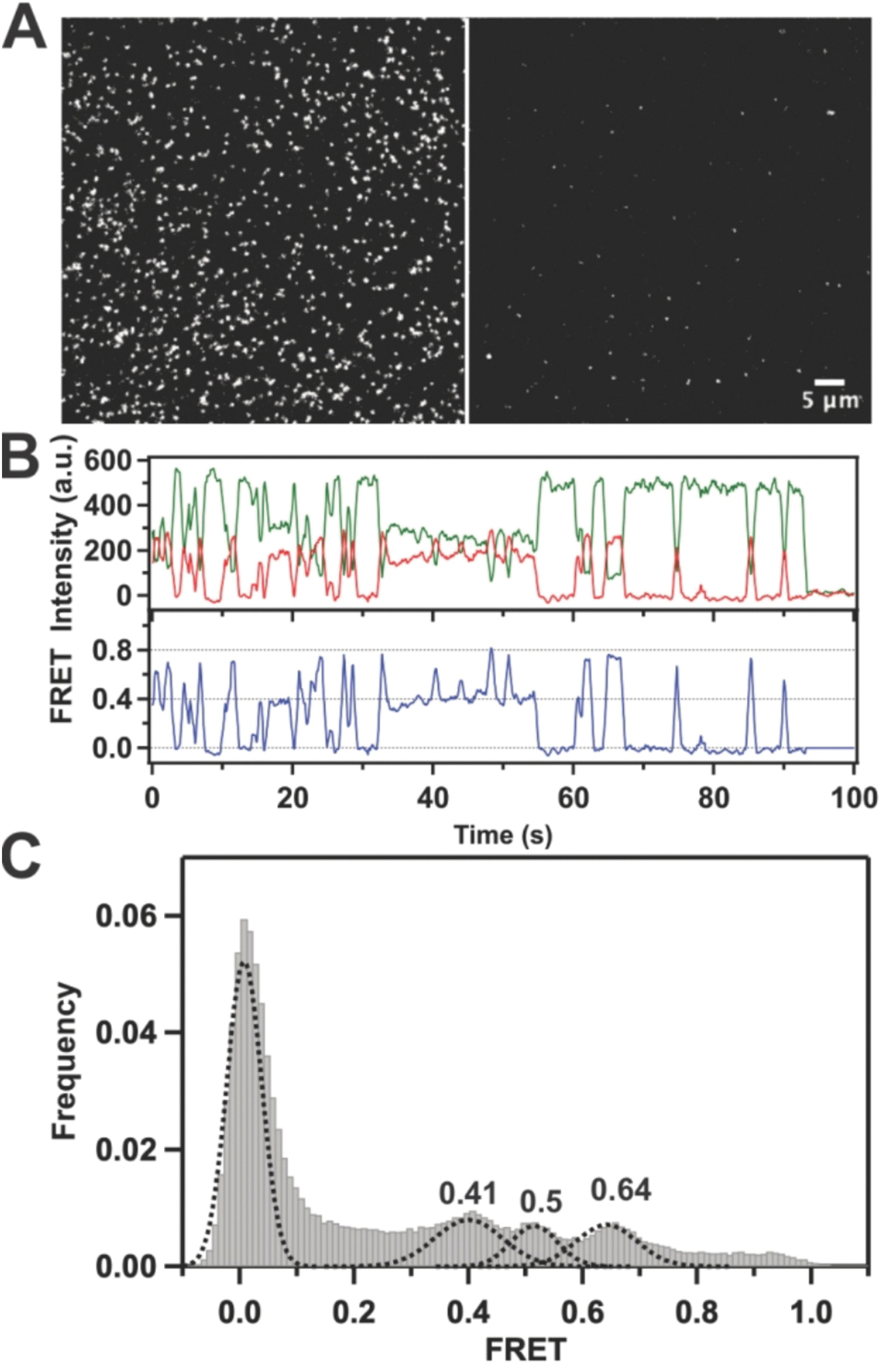
Single-molecule FRET reveals interactions of Klenow Fragment polymerase with template DNA. **(A)** TIRF image representing a binding of acceptor-labeled (20 nM) L744C KF molecules to immobilized donor-labeled DNA molecules (left image) produces fluorescent spots in the acceptor channel (right image). The vertical white line separates the donor (left) and acceptor (right) channels. **(B)** Binary KF complexes exhibit fast sampling of multiple bound states. The green and red traces show background corrected fluorescence intensities in the donor and acceptor channels respectively. The blue line shows corresponding smFRET trajectory. **(C)** The FRET histogram generated from 121 individual smFRET time trajectories (molecules), reveals three major conformations at 0.41, 0.5 and 0.64 when the polymerase is bound to the DNA substrate. These FRET states are represented by black dashed lines representing individual Gaussian distributions. The distribution of smFRET efficiency was identified using non-constrained multi-peak Gaussian function. The peak at 0.0 FRET represents polymerase dissociation from the DNA substrate, where no energy transfer occurs. (For interpretation of the references to colour in this figure legend, the reader is referred to the web version of this article.).

To test the performance of quartz surfaces prepared using 1MP, we conducted several control experiments using biochemical systems that had been characterized previously [25]. *Here*, we analyzed conformational changes of *Escherichia coli* DNA polymerase I (Klenow fragment, KF) when it binds to its substrate DNA molecules immobilized on the microscope surface [25]. In this experiment, KF molecules were fluorescently labeled with Alexa Fluor 594 acceptor dyes attached in the finger’s domain via L744C cysteine substitution. The substrate dsDNA contained a 54-nt template labeled with biotin on its 3′ end and a 17-nt primer labeled with Alexa Fluor 488 donor dye at position 8 from the 3′ terminus. This labeling scheme allows us to monitor conformational changes of the flexible finger domain as polymerases bind to their immobilized DNA substrates and form binary complexes. The labeled DNA and L744C KF samples were generously provided by the Millar Lab at The Scripps Research Institute [25]. Formation of the binary complexes is identified by the appearance of fluorescence spots in the acceptor channel (Fig. 6A, right panel). As a control, we introduced the labeled polymerase into a streptavidin-coated slide chamber without immobilized DNA and illuminated the sample to assess non-specific adsorption. Under these conditions, we quantified 74.3 ± 31.8 (mean ± s.d.) polymerase molecules per field-of-view (Fig. S8, Supplementary Table S3). This was significantly lower than the number of molecules quantified in the presence of donor DNA (Fig. S8, Supplementary Table S3, 629.0 ± 52.6). Individual time traces obtained for immobilized donor molecules (Fig. 6A, left panel) show that polymerases bind to their DNA substrate for relatively short time intervals, dissociate and re-bind again, but not necessarily the same polymerase molecules (Fig. 6B). The finger domain of KF is known to undergo a large structural conformation among several states, with a range of motion spanning ~ 10 Å. Indeed, in our single-molecule FRET experiments, we were able to resolve three conformational states of KF in the binary complexes (Fig. 6C) [25]. The high quality of the fluorescence trajectories in the donor and acceptor channels provided improved resolution of the calculated FRET efficiency, necessary for the separation of the three closely positioned FRET states at 0.41, 0.5, and 0.64.

### 5.2. Quantifying oligomerization of Intact proteins through Single-Molecule fluorescence photobleaching

**Fig. 7.**
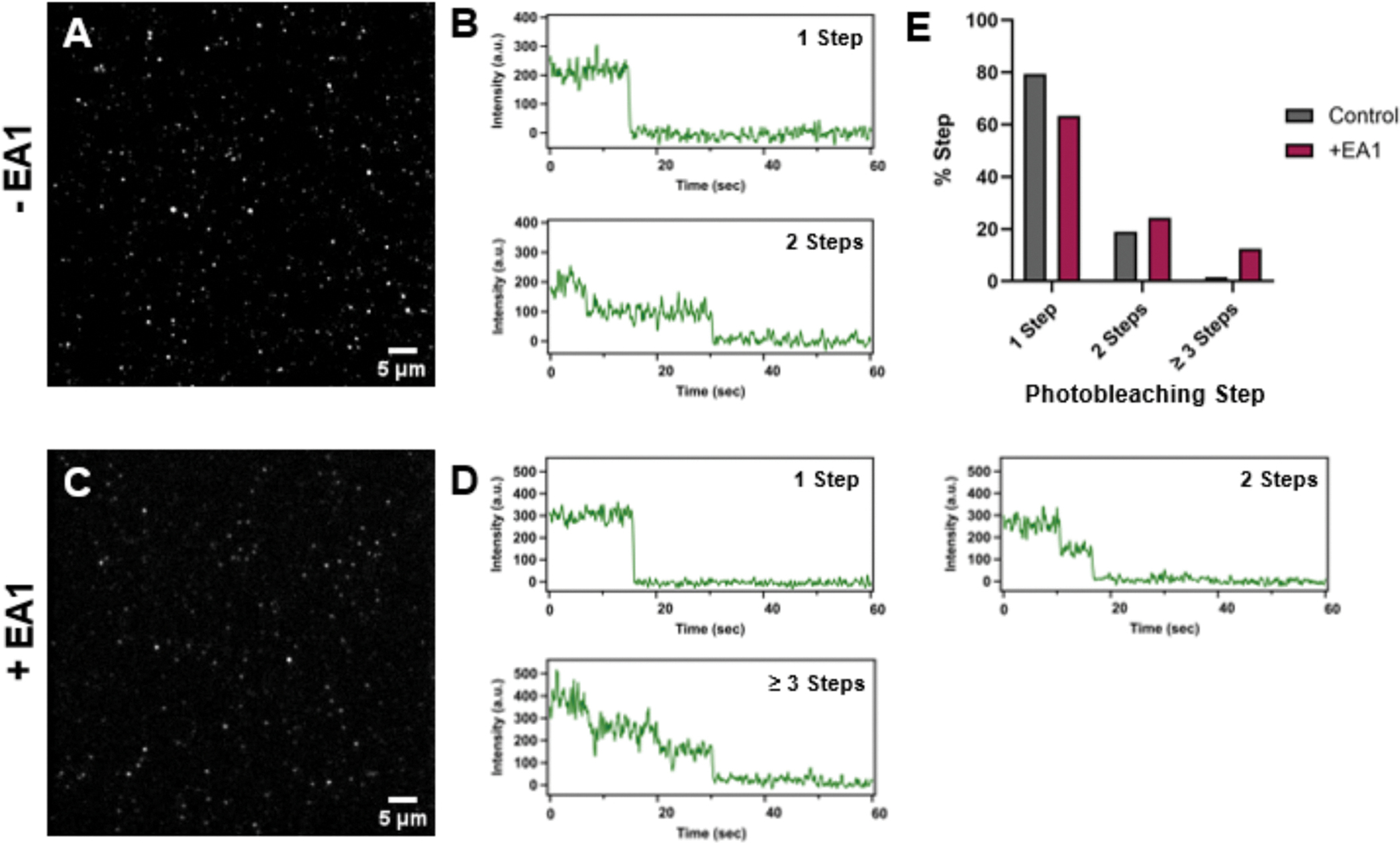
Oligomerization of EphA2. SiMPull-POP results of an EphA2-GFP construct harvested from DU145 mammalian cells. Representative TIRF images of immobilized EphA2-GFP DIBMALPs in control **(A)** and EA1-treated **(C)** conditions (≤5 nM). Examples of 1-, 2-, and ≥ 3 step (EA1-treated only) photobleaching traces of EphA2-GFP in the presence **(D)** and absence **(B)** of its ligand EA1. **(E)** Quantification of photobleaching steps of EphA2-GFP in DIBMALPs with and without ligand treatment. A total of 126 molecules were analyzed for control and 177 in the presence of EA1.

Next, we aimed to test the functionality of the 1MP method for isolating a protein of interest from a cellular context. To do this, we utilized the SiMPull-POP protocol [30] to immobilize a full-length protein of interest. *Here*, we used a C-terminally tagged GFP version of the receptor tyrosine kinase EphA2 that was stably expressed in a modified DU145 mammalian cell line. Cellular extracts were lysed in the absence of detergent so that the protein construct could be recapitulated with native lipids into DIBMALPs (di-isobutylene maleic acid lipid particles). The solubilized cellular lysates were then incubated on 1MP prepared slides that were pre-incubated with NeutrAvidin and a biotinylated EphA2 antibody and washed thoroughly to remove non-specific interactions (Fig. 7A & C). Fluorescent intensity over time of immobilized EphA2-GFP (Fig. 7B) or EphA2-GFP treated with its ligand Ephrin-A1 (EA1) (Fig. 7C & D) were recorded. The percentage of 1-, 2-, and ≥ 3 photobleaching steps in each condition (Fig. 7E) was analyzed as described above. Under control conditions, EphA2 had 79% 1-step, 19% 2-step, and 2% ≥ 3 photobleaching steps. When the cell lysates were treated with EA1, we observed a shift towards EphA2 dimer and oligomer populations (63% 1-step, 24% 2-step, and 12% ≥ 3 steps). Treatment of EphA2 with EA1 induces receptor oligomerization, which is reflected in our data [18,30,45].

## 6. Concluding Remarks

Single-molecule experiments with surface-immobilized biomolecules provide a unique opportunity to study and characterize biomolecular interactions over long timescales. However, non-specific interactions between biomolecules of study, especially protein samples and the slide surface, can complicate experiments and alter molecular behavior. Minimizing these interactions is essential for accurately characterizing biomolecular processes. Surface passivation for these studies is typically achieved using PEG. Although several PEGylation methods exist, they often require elongated incubation times or require specialized equipment. In conclusion, we present a new protocol (1MP) using PEG-Silane that enables rapid PEGylation of microscope slides, resulting in improved surface quality for characterizing biomolecules. Compared to the traditional PEG-SVA method, our 1MP protocol significantly reduces not only the time for slide passivation but also the non-specific interactions of various samples with the slide surface, particularly small proteins or peptides that are otherwise prone to surface adsorption. By uniformly exposing the entire slide surface to the PEG solution through careful dipping, the protocol promotes the formation of a consistent PEG layer and minimizes non-specific sample interactions and adsorption onto the slide. In contrast, traditional PEG-SVA methods rely on placing a coverslip over the solution, which leads to air microbubbles and pockets of non-PEGylated surfaces, ultimately increasing non-specific interactions with the samples. We further demonstrate that the 1MP method is broadly applicable to single-molecule studies, including pull-down assays and the immobilization of conjugated peptides, fluorescent proteins, and labeled full-length proteins for photobleaching and smFRET experiments. Collectively, we present here a facile, efficient, and versatile PEGylation protocol suitable for a wide range of applications in the single-molecule field.

## Supplementary Material

1

## Data Availability

Data will be made available on request.

## Appendix A. Supplementary data

Supplementary data to this article can be found online at https://doi.org/10.1016/j.ymeth.2026.01.003.
