# Supplementary material for: Rapid and improved surface passivation method for Single-Molecule experiments
Source: Methods. Author manuscript; Available in PMC 2026 Feb 25. (PMC12933383; doi:10.1016/j.ymeth.2026.01.003)
Supplement: 1 [file NIHMS2138426-supplement-1.docx]

**Supplemental Material**

**Rapid and Improved Surface Passivation Method for Single-Molecule TIRF Experiments**

Alyssa N. Gonneville^1#^, Alyssa E. Ward^1#^, Narisa Ria Naidoo^1^, Francisco N. Barrera^1^, Rajan Lamichhane^1*^

**A_2A_AR-Cterm Peptide Sequence:**

PFIYAYRIREFRQTFRKIIRSHVLRQQEPFKAAGTSARVLAAHGSDGEQVSLRLNGHPPGVWANGSAPHPERRPNGYALGLVSWGGSAQESQNTGLPDVELLSHELKGVCPEPPGLDDRLAQDGAGVSHHHHHH

**Supplemental Figure 1. SDS-PAGE of Purified A_2A_AR-Cterm Peptide**


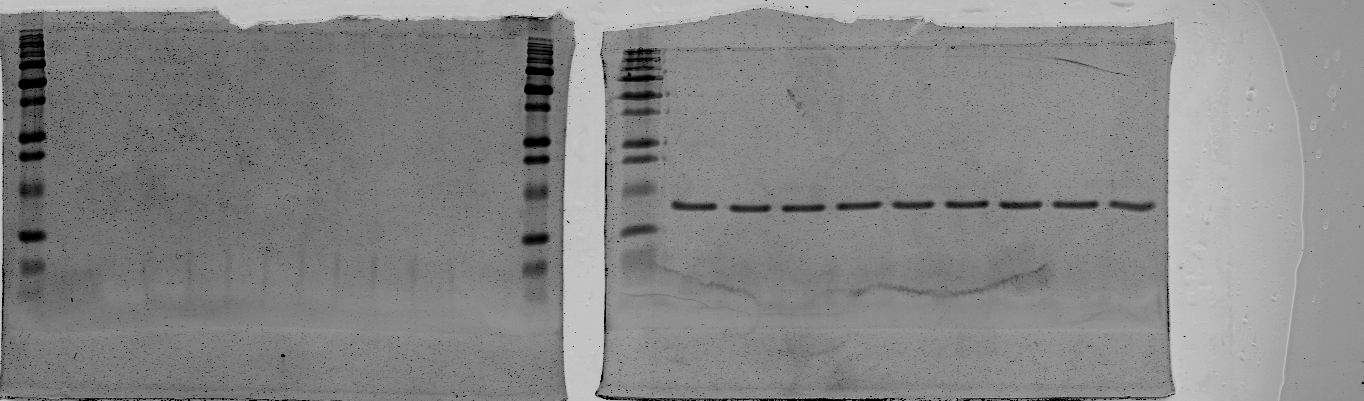


250

150

100

75

50

37

25

20

15

10

5

1 2

SDS-PAGE gel electrophoresis of the purified A_2A_AR-Cterm peptide (Lane 2). The molecular weight of the peptide is expected to be ~14.7 kDa. Lane 1: Protein Marker

**Supplemental Figure 2. Comparison of the number of A_2A_AR-ctL peptide molecules selected by IDL on PEG-SVA functionalized quartz slides incubated with and without streptavidin as the immobilization agent.**

**
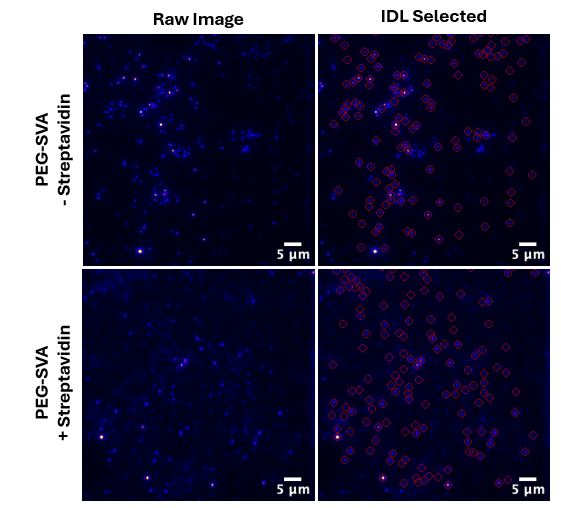
**

Comparison of the number of selected molecules determined by the IDL script from the raw .TIF image generated from the video recording. The A_2A_AR-Cterm peptide (125 nM) was incubated on a PEG-SVA functionalized quartz slide for 15 minutes in the presence and absence of streptavidin.

**Supplemental Figure 3. Comparison of the number of A_2A_AR-ctL peptide molecules selected by IDL on PEG-Silane functionalized quartz slides incubated with and without streptavidin as the immobilization agent.**


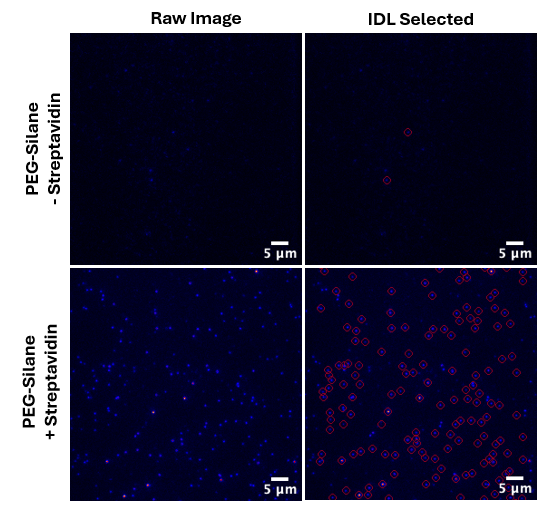


Comparison of the number of selected molecules determined by the IDL script from the raw TIF image generated from the video recording. The A_2A_AR-Cterm peptide (125 nM) was incubated on a PEG-Silane functionalized quartz slide for 15 minutes in the presence and absence of streptavidin.

**Supplemental Figure 4. Quantification of the number of molecules on PEG-SVA and PEG-Silane functionalized slides incubated with the C-terminal peptide.**


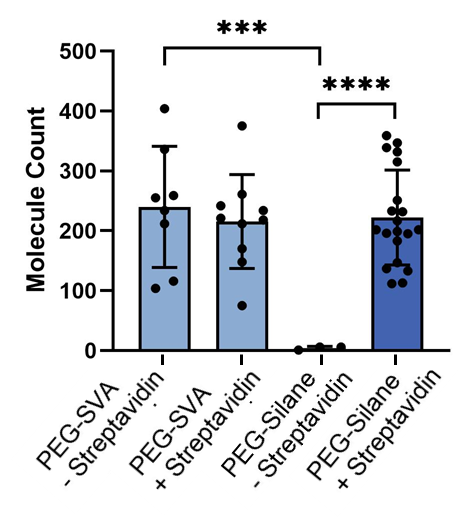


Comparison of the number of molecules selected on PEG-SVA and PEG-Silane functionalized quartz slides incubated with the C-terminal peptide in the presence and absence of streptavidin (immobilization agent). An unpaired *t*-test was run for statistical analysis between paired (PEG-SVA vs PEG-Silane) conditions (+/+ and -/- streptavidin). ****p*-value = 0.0003, *****p*-value = <0.0001

**Supplemental Figure 5. TIRF images of Alexa Fluor555 Anti-mouse on 1MP prepared slides in the presence and absence of immobilization agent.**

**
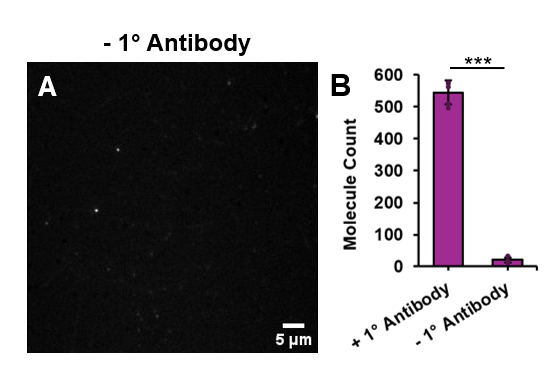
**

**(A)** Representative TIRF image of Alexa Fluor 555 conjugated mouse antibody (250 pM) incubated in the absence of biotinylated primary mouse anti-histidine antibody on a 1MP prepared slide (slide was incubated initially with streptavidin). **(B)** Number of molecules selected per field-of-view on slides incubated with Alexa Fluor 555 in the presence and absence of the primary antibody. Bars represent the mean ± standard deviation. Dots represent individual fields of view quantified (five per condition shown). ****p*-value = 6.76 x 10^-6^, an unpaired *t*-test was run for statistical analysis.


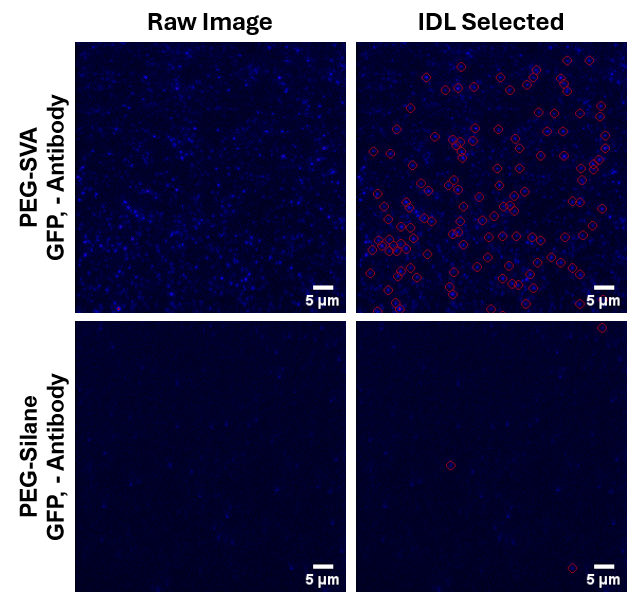
**Supplemental Figure 6. Comparison of the number of molecules selected by IDL on PEG-SVA and PEG-Silane functionalized quartz slides incubated with free GFP.**

Comparison of the number of selected molecules determined by the IDL script from the raw .TIF image generated from the video recording. Sample is 15 nM free GFP incubated on either a PEG-SVA or PEG-Silane functionalized quartz slide for 30 minutes in the absence of anti-GFP (immobilization agent).

**Supplemental Figure 7. TIRF microscopy of YFP on 1MP prepared slides in the presence and absence of antibody immobilization.**

**
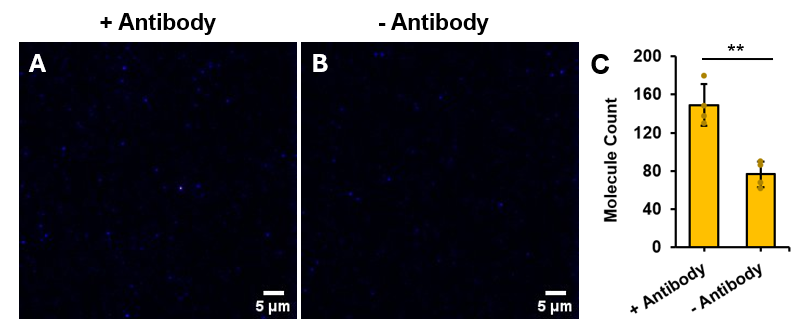
**

**(A)** TIRF image of YFP immobilized by a biotinylated GFP antibody on a 1MP prepared slide. **(B)** YFP incubated on a 1MP prepared slide in the absence of the immobilization antibody (both slides were incubated initially with NeutrAvidin). **(C)** Number of molecules selected per field-of-view (four fields of view quantified per condition). Bars represent the mean ± standard deviation. Dots represent individual field-of-view quantification point. ***p*-value = 0.0025, an unpaired *t*-test was run for statistical analysis.

**Supplemental Figure 8. TIRF imaging of DNA polymerase KF on 1MP prepared slides in the presence and absence of donor DNA.**


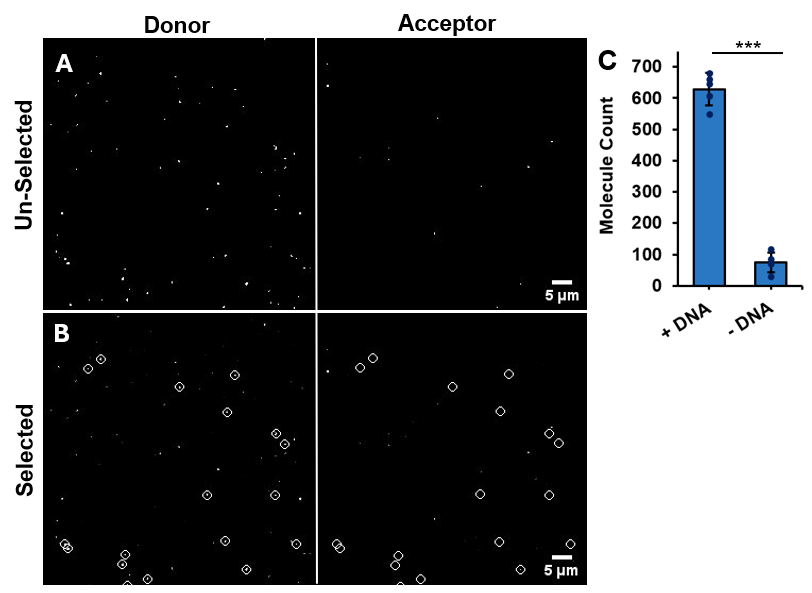


Representative TIRF images of Alexa Fluor 594-labeled DNA polymerase KF (20 nM) on the surface in the absence of immobilized DNA, excited at 465 nm **(A),** with the molecules selected by IDL **(B)**. Quantification of the number of molecules selected per field-of-view of the DNA polymerase sample in the presence (refer to main text Figure 6A) and absence of donor DNA **(C)**. Bars represent the mean ± standard deviation. Dots represent individual fields of view (5 shown here). ****p*-value = 1.84 x 10^-7^, an unpaired *t*-test was used for statistical analysis.

**Supplemental Table 1. Comparison of different slide Cleaning and PEGylation protocols**

| **Paper** | **Cleaning Incubation Time** | **Cleaning Steps** | **PEG Solution/ Incubation Time** | **Protocol Considerations** |
| --- | --- | --- | --- | --- |
| [1] | 85+ min. | 1. Water rinse x3 times, 5 min. Sonication, rinse x3 times 2. 20 min. sonication in acetone 3. Water rinse x3 times 4. 20+ min. sonication in 1 M potassium hydroxide (KOH) 5. Water rinse x3 times 6. 20 min. incubation in Piranha solution (3:1 ratio of sulfuric acid (H_2_SO_4_) to hydrogen peroxide (H_2_O_2_) 7. Water rinse x3 times 8. Methanol rinse x1 time 9. Aminosilanization with APTES (3-aminopropyl trimethoxysilane), methanol (MeOH), and acetic acid, 20-30 min with 1 min. sonication 10. Methanol rinse x1 time 11. Dry with an inert gas (Ar or N_2_) 12. Incubate with PEG solution for a minimum of 2 hours to overnight | **PEG type/concentrations:**  1.07 mM NHS-ester mPEG (5000 Da, Laysan Bio.) / 26.7 µM biotinylated NHS-ester PEG (5000 Da, Laysan Bio.)  **Solution:**  Prepared in a 100 mM sodium bicarbonate buffer (pH 8.5), centrifuged before addition to the surface  **Incubation time:**  2 hours to overnight | 1. Requires a humidity chamber and overnight incubation for PEGylation, with the need to place a coverslip on top of a slide for passivation of both surfaces  2. A second PEGylation step is suggested with 25 mM MS4-PEG (NHS-ester PEG molecules (333 Da)) for pulldown experiments  3. Confirmed storage up to three months  4. Protocol written for 5-15 slides/coverslips, but does not specify steps for regeneration of slides  Hazardous waste: KOH, MeOH, H_2_SO_4_ / H_2_O_2_, APTES |
| [2] | 158 min. | 1. Boil in a diluted 7x cleaning solution for 30 min. 2. Rinse with water 3. Rinse with isopropyl alcohol (IPA) 4. Dry with an inert gas (Ar or N_2_) 5. **Coated with silicon dioxide (SiO_2_) by plasma-enhanced chemical vapor deposition (PECVD) or spin-coated with hydrogen silsesquioxane (HSQ), then cured at 540 °C for 2 hours. 6. Incubate 3 minutes in Piranha solution (3:1 ratio H_2_SO_4_ / H_2_O_2_) that was prepared and allowed to sit for 1.5 hours prior to sample insertion 7. Clean with oxygen plasma for 5 minutes 8. PEGylate samples in solution for 48 hours | **PEG type/concentrations:**  ~16 µM mPEG-Silane (5000 Da, Laysan Bio.)  **Solution:**  Prepared in anhydrous toluene with acetic acid (~20.8 mM)  **Incubation time:**  48 hours | 1. Shortened cleaning procedure compared to other protocols but requires access to plasma cleaning equipment, PECVD instrumentation (if needed), and equipment to incubate slides/coverslips at high temperature (540 °C)  2. Extended PEGylation time (48 hours) and uses toluene as the solvent  Hazardous waste: IPA, HSQ, H_2_SO_4_ / H_2_O_2_, toluene, acetic acid, and methyl-isobutyl ketone  ** Procedure included an additional coating for the coverslips for experimental needs; if not needed, the cleaning step could be reduced to a 2-hour incubation at 540 °C. |
| [3] | 125 min. | 1. Sonication in Piranha solution (3:1 ratio H_2_SO_4_ / H_2_O_2_) for 90 min at 20-30°C 2. Sonication in 0.5 M sodium hydroxide (NaOH) for 30 min. 3. Sonication in HPLC-grade acetone for 5 min. 4. Dry with an inert gas (Ar or N_2_) 5. Preheat the passivation surface at 90 °C for 5 min. in a reaction chamber (see paper for details) 6. Incubation with PEG solution at 90 °C for 15 min. in the reaction chamber | **PEG type/concentrations:**  99:1 ratio w/w mPEG-Silane (5000 Da, Laysan Bio.) / biotin-PEG-Silane (5000 Da, Laysan Bio.)  or 25% (w/w) mixture of mPEG-Silane  **Solution:**  Anhydrous DMSO  **Incubation time:**  15 minutes + 5 minutes of preheating the surface for passivation | 1. Requires a custom reaction chamber with a placeholder for the coverslip that can be heated to a high temperature. Additionally, a silicon mold is used to isolate the PEG solution on the coverslip, both of which limits the number and area of surfaces that can be functionalized at one time.  2. Tested for glass coverslips, further test with slides and their regeneration will need to be explored.  3. Several time points were tested with a 15 min. incubation time, showing the lowest non-specific background binding with a small conjugated sample  Hazardous waste: H_2_SO_4_ /H_2_O_2_, NaOH, acetone, DMSO |
| [4] | 60-90+ min.  (with an overnight incubation plus 60-100 min. for pre-used slides) | 1. *Overnight immersion in methanol and acetone* 2. *Boil for 5–10 min. in water* 3. *Sonication in 5% Alconox for 20–30 min (scrub after)* 4. *Sonication in water for 20–30 min.* 5. *Pass through flame for 30+ sec.* 6. *Sonication in water for 20–30 min.* 7. Incubated in 1 M KOH for 30–45 min. 8. Pass through flame for 2–3 min. (slides only, pass coverslips x4-5 times through the flame only) 9. Incubate in aminosilane for 30–45 min. 10. Incubated for 4 hours to overnight with PEG solution 11. Rinse with water 12. Dry with an inert gas (Ar or N_2_) | **PEG type/concentrations:**  98% mPEG (5000 Da, Laysan Bio.) / 2% biotin-PEG (5000 Da, Laysan Bio.)  **Solution:**  **Incubation time:**  4 hours to overnight | 1. Requires heating the surfaces by flame, but does not require additional instrumentation for cleaning  2. Includes 1-2 overnight incubations  3. Longer PEGylation step (4+ hours)  Hazardous waste: Acetone, KOH, aminosilane |
| [5] | 90-115+ min.  (Plus 5 hours and 5-10 min. for pre-used slides) | 1. *5-hour immersion in ethanol or acetone* 2. *Boil in water* 3. *Sonication in 5% (w/v) Alconox detergent for 5–10 min.* 4. *Rinse with water and scrub the surface* 5. *Pass through flame (expose both sides)* 6. *Cool surfaces with inert gas* 7. Sonication in 1 M KOH for 30-45 min. 8. Sonication in water for 20–30 min. followed by a final rinse in water 9. Pass through flame till dry and cool with inert gas 10. Dry coverslips with nitrogen gas, burn 5–6 times with quick passes through the flame and put another dry jar. 11. Rinse in methanol once 12. Incubation (absence of light) in methanol with 1.3% glacial acetic acid and 1.3% N-(2-aminoethyl)-3-aminopropyltrimethoxysilane for 10 min., sonication for 1 min, then incubate for another 30 min. 13. Thorough rinse with water 14. Dry with an inert gas (Ar or N_2_) 15. Incubate with PEG solution for 3-5 hours in the absence of light. | **PEG type/concentrations:**  40-42.2μM mPEG-SVA (5000 Da, Laysan Bio.) / 0.44-0.67 μM Biotin-PEG-SVA (5000 Da, Laysan Bio.)  **Solution:**  Prepared in a 100 mM sodium bicarbonate buffer, centrifuged before addition to the surface  **Incubation time:**  3-5 hours | 1. Requires multiple rounds of burning the slides/coverslips by flame. Extensive heating (by flame), especially coverslips, can damage or cause breaking of the slides/coverslips  2. Includes regeneration and functionalization incubations of 180-300 min.  3. Protocol written for 12 slides  4. A second PEG/passivation step is suggested to improve quality, especially for pulldown assays  Hazardous waste: Ethanol, KOH, acetic acid, APTES |
| [6] | 102 min. plus an overnight incubation | 1. Sonication in 20% (v/v) Extran MA 01 at 50°C and 100% power for 20 min. 2. Rinse with water 3. Sonication in water at 50°C, 100% power for 20 min. 4. Rinse with water 5. Incubate overnight in 10% hydrochloric acid (HCl) 6. Rinse with water 7. Incubation in Piranha solution (30% hydrogen peroxide: 95% sulfuric acid 1:2) for 1 hour 8. Rinse with water 9. Dry with an inert gas (Ar or N_2_) 10. PEGylation was performed in dry toluene overnight at 80°C in a reaction flask* under nitrogen, see paper for details. 11. Cool to room temperature, rinse in ethyl acetate x3 times 12. Rinse in methanol x3 times 13. Sonication in methanol for 2 min. at 100% 14. Dry with an inert gas (Ar or N_2_) | **PEG type/concentrations:**  0.5 mg/mL mPEG2000-urea-triethoxysilane (synthesized as in Blümmel *et al*, 2007)  **Solvent:**  Dry toluene + triethylamine  **Incubation time:**  Overnight | 1. Requires a specialized reaction vessel with a nitrogen atmosphere at 80°C  2. Requires an overnight incubation in HCl  3. Protocol written for a batch of 4 coverslips  4. Storage reported at 1 week before use  Hazardous waste: HCl, sulfuric acid/hydrogen peroxide, toluene, ethyl acetate, methanol |
| [7] | 93 min. | 1. Incubate in Piranha solution for 30 min. 2. Rinse with water, sonicate in water for 3 min. then rinse with water once 3. Incubation in 3 Glycidyloxypropyl)-trimethoxysilane (GOPTES) for 1 hour under a nitrogen atmosphere. 4. Rinse with dry cyclohexane 5. Dry with an inert gas (Ar or N_2_) 6. (A) Incubated with PEG solution at 75–95 °C for 40–48 hours or (B) in dry 0.25mM toluene/μM triethylamine for 16–20 h at 80 °C under a nitrogen atmosphere. 7. (A) Rinse with water for several hours or (B) Rinse with ethyl acetate, then sonicate for 2 min., rinse once with ethyl acetate, then methanol 8. Dry with inert gas (Ar or N_2_) | **PEG type/concentrations:**  mPEG derivatives (for details, see paper)  **Solvent:**  3-isocyanatopropyltriethoxysilane (3-ITPS), dioxane, dimethylformamide  **Incubation time:**  (A) 40-48 hours or (B) 16-20 hours | 1. Requires a reaction vessel with a nitrogen atmosphere at high temperature  2. Offers a shortened cleaning procedure, but an elongated PEGylation step  Hazardous waste: GOPTES, cyclohexane, toluene, triethylamine, ethyl acetate, methanol, and Piranha |
| [8] | 100+ min. | 1. *Scrub surfaces with Alconox paste* 2. *Rinse the detergent off with water* 3. Rinse with ethanol, then water 4. Boil the slides for 20+ min. 20% NH_4_OH and 30% H_2_O_2_ 5. Rinse with water 6. Pass through the flame to dry 7. Rinse with water 8. Sonication in 1 M KOH for 30 min. 9. Rinse with water, then sonicate for 30 min. in methanol 10. Incubate (in the absence of light) in a solution of 5% acetic acid, 1% 3-aminopropyltriethoxysilane in methanol for 10 min., then sonicate for 1 min, then incubate for 10 min. 11. Rinse once with methanol, then water, and once again with methanol. 12. Dry with an inert gas (Ar or N_2_) 13. Incubate with PEG solution overnight in a humidity chamber in the dark 14. Rinse with water 15. Dry with an inert gas (Ar or N_2_) | **PEG type/concentrations:**  ~50 μM mPEG-succinimidyl carboxymethyl (mPEG-SCM, 5000 Da, Laysan Bio.) / 15.6-25 mg/mL Biotin-PEG-SCM (3400/5000 Da, Laysan Bio.)  **Solution:**  Prepared in centrifuged sterile filtered (0.2 μm syringe filter) 100 mM sodium bicarbonate (NaHCO_3_, pH 8.4)  **Incubation time:**  Overnight | 1. Protocol written for five slides/coverslips  2. Requires an overnight incubation in a humidity chamber with coverslip/slide pairs incubated with the PEG solution layered between  3. Requires flaming slides/coverslips  4. Can be stored for up to 2 weeks  Hazardous waste: Ethanol, ammonium hydroxide/hydrogen peroxide, KOH, methanol, acetic acid, and APTES |
| [9] | 137 min.  (Plus 75 min. for pre-used slides) | 1. *Boil in water for 15 min.* 2. *Sonication in 20% Contrad 70 for 20 min.* 3. *Rinse with water x3* 4. *Sonication in water for 40 min.* 5. Incubate in 20% ammonium hydroxide, 20% hydrogen peroxide for 15 min. 6. Rinse with water 7. Dry by flame 8. Sonication in 1 M KOH for 20 min. 9. Rinse with water, then sonicate in water for 40 min. 10. Sonicate in methanol for 40 min. 11. Rinse with water x3 12. Dry with an inert gas (Ar or N_2_) 13. Incubate in 2-3% APTES at 20°C–23°C for 10 min. 14. Sonicate in the solution for 2 min. then incubate in the solution again for 10 min. 15. Rinse with methanol, then water, then methanol again. 16. Dry with an inert gas (Ar or N_2_) 17. Incubate in the absence of light under a humidity chamber for 12-14 hours. 18. Rinse with water 19. Dry with an inert gas (Ar or N_2_) | **PEG type/concentrations:**  40 μM mPEG-SVA (5000 Da, Laysan Bio.) /2.5 μM Biotin-PEG-SVA (5000 Da, Laysan Bio.)  **Solution:**  Prepared in sterile filtered (0.22-micron filter) 100 mM sodium bicarbonate solution, centrifuged  **Incubation time:**  12-14 hours | 1. Protocol written for 5 slides, but is stable in storage for 3-4 weeks  2. Requires an overnight incubation in a humidity chamber with PEG solution added to the slide and coverslip layered on top of the solution  3. Requires flaming slides/coverslips  Hazardous waste: Ammonium hydroxide/hydrogen peroxide, KOH, methanol, acetic acid, and APTES |
| 1MP | 80 min.  (Plus 75 min. for pre-used slides) | 1. *Boil in water for 15 min.* 2. *Sonicate in 20% Contrad 70 for 20 min.* 3. *Rinse with water x3 times.* 4. *Sonicate in water for 40 min.* 5. Sonicate in 1 M KOH for 20 min. 6. Rinse with water x3 times, 7. Sonicate in water for 40 min. 8. Incubate in Piranha solution (3:1 ratio 75% H_2_SO_4_ and 25% H_2_O_2)_ for 20 min. 9. Rinse with water x3 times, then with methanol once 10. Prepare a PEG solution, dip the surfaces into the solution for 1 minute, then rinse in acetone for 30 seconds. 11. Dry them with an inert gas (Ar or N_2_) | **PEG type/concentrations:**  3.9-4.9 µM mPEG-Silane (Laysan Bio., Inc., 5,000 Da) / 0.2-0.4 µM of Biotin-PEG-Silane (Laysan Bio., Inc., MW 5,000)  **Solution:**  Prepared in a 1:1 acetone/methanol mix, then diluted into a volume (200mL) of acetone  **Incubation time:**  1 minute plus 30 sec rinse | 1. Offers a short PEGylation step  2. Can be used for the regeneration of slides  3. Slides/coverslips can be stored for at least a month  4. Does not require heating slides/coverslips  Hazardous waste: Sulfuric acid/hydrogen peroxide, KOH, acetone, and methanol |

**Italicized text represents cleaning steps for the regeneration of pre-used slides*

*Note: Storage of slides at -20°C under inert gas, sealed with parafilm in a 50mL Falcon tube and placed in a desiccant-containing container, was similarly reported in the method that specified storage conditions.*

**Supplementary Table 2:** **Description of the number of Fields of View and molecules per sample and condition analyzed for comparison of PEGylation methods in the absence or presence of immobilization agent.**

| *PEG* | *Amount** | *Price** |
| --- | --- | --- |
| mPEG-SVA | 1g, 5g | $140, $480 |
| Biotin-PEG-SVA | 100mg, 500mg, 1g | $200, $295, $400 |
| mPEG-SVA/biotin-PEG-SVA | 1g/100mg | $300 |
| mPEG-Silane | 1g, 5g | $100, $300 |
| Biotin-PEG-Silane | 500mg, 1g | $290, $400 |

**Prices and currently available reagent amounts are reported from Laysan Bio. Inc. at the time of publication, both are subject to change.*

**Supplementary Table 3:** **Description of the number of fields-of-view and molecules analyzed per sample and condition for comparing PEGylation methods in the absence or presence of an immobilization agent.**

| Sample | Condition | Fields of View Recorded | Number of Molecules/Field of View | Mean | Standard Deviation |
| --- | --- | --- | --- | --- | --- |
| C-terminal Peptide | PEG-SVA/ No Streptavidin | 8 | 259, 104, 116, 212, 234, 255, 404, 336 | 240.0 | 100.7 |
|  | PEG-SVA/ With Streptavidin | 10 | 221, 234, 107, 148, 261, 212, 375, 75, 242, 218 | 209.3 | 84.5 |
|  | PEG-Silane/No Streptavidin | 3 | 1, 6, 6 | 4.3 | 2.9 |
|  | PEG-Silane/With Streptavidin | 20 | 202, 315, 347, 359, 251, 332, 339, 217, 119, 183, 233, 196, 147, 195, 137, 133, 112, 113, 202, 232 | 218.2 | 82.3 |
| Alexa Fluor 555 Anti-Mouse | PEG-Silane/No Primary Antibody | 5 | 13, 13, 18, 19, 33 | 19.2 | 8.2 |
|  | PEG-Silane/With Primary Antibody | 5 | 563, 578, 511, 498, 574 | 544.8 | 37.5 |
| DNA Polymerase | PEG-Silane/No DNA | 5 | 30, 118, 70, 70, 86 | 74.8 | 31.8 |
|  | PEG-Silane/With DNA | 5 | 681, 548, 661, 647, 608 | 629.0 | 52.6 |
| YFP | PEG-Silane/With Antibody | 4 | 130, 138, 148, 180 | 149 | 21.9 |
|  | PEG-Silane/No Antibody | 4 | 62, 86, 90, 68 | 76.5 | 13.6 |
| GFP | PEG-Silane/With Antibody | 7 | 74, 83, 36, 54, 114, 38, 58 | 65.3 | 27.5 |
|  | PEG-Silane/No Antibody | 6 | 7, 20, 14, 8, 11, 1 | 10.2 | 6.5 |
|  | PEG-SVA/No Antibody | 5 | 97, 20, 67, 270, 165 | 123.8 | 97.2 |

**References**:

[1] S.D. Chandradoss, A.C. Haagsma, Y.K. Lee, J.H. Hwang, J.M. Nam, C. Joo, Surface passivation for single-molecule protein studies, J Vis Exp (86) (2014).

[2] H. Cai, S.J. Wind, Improved Glass Surface Passivation for Single-Molecule Nanoarrays, Langmuir 32(39) (2016) 10034-10041.

[3] Y. Gidi, S. Bayram, C.J. Ablenas, A.S. Blum, G. Cosa, Efficient One-Step PEG-Silane Passivation of Glass Surfaces for Single-Molecule Fluorescence Studies, ACS Appl Mater Interfaces 10(46) (2018) 39505-39511.

[4] T. Paul, T. Ha, S. Myong, Regeneration of PEG slide for multiple rounds of single-molecule measurements, Biophys J 120(9) (2021) 1788-1799.

[5] T. Paul, S. Myong, Protocol for generation and regeneration of PEG-passivated slides for single-molecule measurements, STAR Protoc 3(1) (2022) 101152.

[6] A. Sauter, G. Richter, A. Micoulet, A. Martinez, J.P. Spatz, S. Appel, Effective polyethylene glycol passivation for the inhibition of surface interactions of peripheral blood mononuclear cells and platelets, Biointerphases 8(1) (2013) 14.

[7] J. Blummel, N. Perschmann, D. Aydin, J. Drinjakovic, T. Surrey, M. Lopez-Garcia, H. Kessler, J.P. Spatz, Protein repellent properties of covalently attached PEG coatings on nanostructured SiO(2)-based interfaces, Biomaterials 28(32) (2007) 4739-47.

[8] R. Lamichhane, A. Solem, W. Black, D. Rueda, Single-molecule FRET of protein-nucleic acid and protein-protein complexes: surface passivation and immobilization, Methods 52(2) (2010) 192-200.

[9] N. Thakur, S. Wei, A.P. Ray, R. Lamichhane, M.T. Eddy, Production of human A2AAR in lipid nanodiscs for 19F-NMR and single-molecule fluorescence spectroscopy, STAR Protocols 3(3) (2022) 101535.
